# Xpf and Not the Fanconi Anaemia Proteins or Rev3 Accounts for the Extreme Resistance to Cisplatin in *Dictyostelium discoideum*

**DOI:** 10.1371/journal.pgen.1000645

**Published:** 2009-09-18

**Authors:** Xiao-Yin Zhang, Judith Langenick, David Traynor, M. Madan Babu, Rob R. Kay, Ketan J. Patel

**Affiliations:** Medical Research Council, Laboratory for Molecular Biology, Cambridge, United Kingdom; University of Washington, United States of America

## Abstract

Organisms like *Dictyostelium discoideum*, often referred to as DNA damage “extremophiles”, can survive exposure to extremely high doses of radiation and DNA crosslinking agents. These agents form highly toxic DNA crosslinks that cause extensive DNA damage. However, little is known about how *Dictyostelium* and the other “extremophiles” can tolerate and repair such large numbers of DNA crosslinks. Here we describe a comprehensive genetic analysis of crosslink repair in *Dictyostelium discoideum*. We analyse three gene groups that are crucial for a replication-coupled repair process that removes DNA crosslinks in higher eukarya: The Fanconi anaemia pathway (FA), translesion synthesis (TLS), and nucleotide excision repair. Gene disruption studies unexpectedly reveal that the FA genes and the TLS enzyme Rev3 play minor roles in tolerance to crosslinks in *Dictyostelium*. However, disruption of the Xpf nuclease subcomponent results in striking hypersensitivity to crosslinks. Genetic interaction studies reveal that although Xpf functions with FA and TLS gene products, most Xpf mediated repair is independent of these two gene groups. These results suggest that *Dictyostelium* utilises a distinct Xpf nuclease-mediated repair process to remove crosslinked DNA. Other DNA damage–resistant organisms and chemoresistant cancer cells might adopt a similar strategy to develop resistance to DNA crosslinking agents.

## Author Summary

Organisms are constantly exposed to environmental and endogenous molecules that chemically modify the DNA in their genomes. A particularly pernicious chemical modification is when the two strands of DNA are crosslinked. These crosslinks must be removed so that genomes can be copied, and the damage caused by their persistence is often exploited in cancer chemotherapy. It is also no surprise that all organisms have developed effective means to remove these lesions, and work in prokaryotes and eukaryotes has shown that crosslinks are removed by the concerted action of certain DNA repair pathways. Whilst the obvious route of accumulating crosslinks is by exposure to anti-cancer drugs, these lesions may also arise spontaneously in DNA. This could be why inherited inactivation of one of the crosslink repair pathways results in the catastrophic human illness Fanconi anaemia. Here we determine how the social amoeba *Dictyostelium discoideum*, an organism that is unusually resistant to DNA-damaging agents, removes crosslinks. Our results indicate that this organism has evolved a distinct strategy to remove these lesions. More specifically, we discover that a particular nuclease subcomponent removes the crosslinks by a distinct repair process. We postulate that this strategy to remove crosslinks could be used by other DNA damage–resistant organisms and also by cancer cells that have developed resistance to chemotherapy.

## Introduction

DNA interstrand crosslinks are complex lesions that covalently link the two complementary strands of DNA. Agents that cause this type of lesion can originate from an endogenous source such as reactive species generated by lipid peroxidation, or as a consequence of exposure to exogenous mutagens [1]–[5]. For this reason the cytotoxicity of DNA crosslinks is exploited in cancer chemotherapy, where drugs such as cisplatin, mitomycin C and melphalan are administered as potent DNA crosslinking agents. DNA crosslinks are extremely cytotoxic because they form an absolute barrier to replication [6]. In addition, a crosslink present in a gene coding sequence, will also block transcription. Apart from cell death, DNA crosslinks can also lead to cell senescence and dysfunction [7],[8]. These features are observed in humans born with defective crosslink repair as such individuals exhibit growth retardation, stem cell attrition and symptoms consistent with premature aging [9]. These phenotypic features may be due to the accumulation of unrepaired crosslinks in genomic DNA

Crosslinks can also form between adjacent bases on the same DNA strand, which are referred to as intrastrand crosslinks. Of the two classes of crosslinks, interstrand crosslink is believed to be the more cytotoxic. Crystal structures of lesions formed by reacting cisplatin with DNA have now been solved showing that these lesions cause substantial helix distortion. In terms of DNA repair, genetic and biochemical studies have shown that intrastrand crosslinks are largely repaired by nucleotide excision repair [10]. Repair of interstrand crosslinks is much more complex and poorly understood. Much of the work here is underpinned by genetic studies of classes of mutants that in certain organisms render cells selectively or generally sensitive to chemical crosslinking agents. Four clear repair gene groups in vertebrates stand out in this manner: the Fanconi anaemia (FA) genes, the translesion DNA polymerases Rev1 and Rev3, homologous recombination (HR) repair genes and finally the structure-specific nucleases subcomponents XPF and Mus81 [11]–[13]. Taking this knowledge into account a replication-coupled model for interstrand crosslink repair has been proposed. This model suggests that replication pausing at or near a crosslink initiates a cleavage (a step commonly referred to as unhooking), which is followed by lesion bypass over the crosslinked base by translesion DNA synthesis (TLS). An intact chromatid is therefore created and can now be used as a template to complete repair by HR [11],[13].

Not all the gene groups that function in vertebrate crosslink repair are conserved in yeast. Apart from FANCM none of the other 12 Fanconi anaemia genes appears to have orthologues in this organism [14],[15]. This limits the use of yeast in understanding crosslink repair in higher eukaryotes. Crosslink repair has therefore been largely studied in immortalised vertebrate cell lines (such as chicken DT40 cells or Chinese hamster ovary cells). A drawback of some of these systems is however that they contain mutations in other genes such as *p53* that may influence repair. For these reasons some workers have turned to worms and flies [16],[17], as both organisms are genetically tractable and have some of the vertebrate crosslink repair groups conserved. A potential limitation of these model systems is that they are multicellular organisms and consequently DNA repair cannot be easily studied at the level of a single cell. All these factors led us to develop *Dictyostelium discoideum* as a new model for eukaryotic crosslink repair.

*Dictyostelium* is a simple, soil-dwelling organism, which under optimal growth conditions exists as a unicellular amoeba, feeding on bacteria and dividing by binary fission. However, upon starvation, a precisely regulated developmental program is triggered, leading individual amoebae to aggregate and form a multicellular fruiting body [18]. *Dictyostelium* is easy to culture as axenic strains can be grown under standard laboratory conditions [19]. It possesses a small, compact genome that is fully sequenced [20], thereby greatly facilitating genomic and bioinformatics analyses. In addition to this, the organism is genetically tractable as it is straightforward to knock genes out [21],[22] and to carry out random mutagenesis screens [23],[24]. However, an unusual feature of *Dictyostelium* is that it is highly resistant to DNA-damaging agents. Significant numbers of cells can survive exposure to 300 kilorads of ionising radiation, a striking observation that makes *Dictyostelium* one of the most radioresistant organisms known and places it on par with *Deinococcus radiodurans* [25]. This resistance is not just restricted to radiation. *Dictyostelium* also shows resistance to UV light [26] and to many chemical mutagens [27], some of which are produced by bacteria in the soil [28]. Highly efficient DNA repair responses might therefore have evolved in *Dictyostelium* to enable it to survive in such a highly mutagenic environment. We believe that studying how this organism responds to DNA crosslinks provides us with a unique opportunity to see how a DNA damage resistant organism can deal with these important lesions.

## Results

### Disruption of the FA pathway results in moderate sensitivity to cisplatin

**Figure 1:**
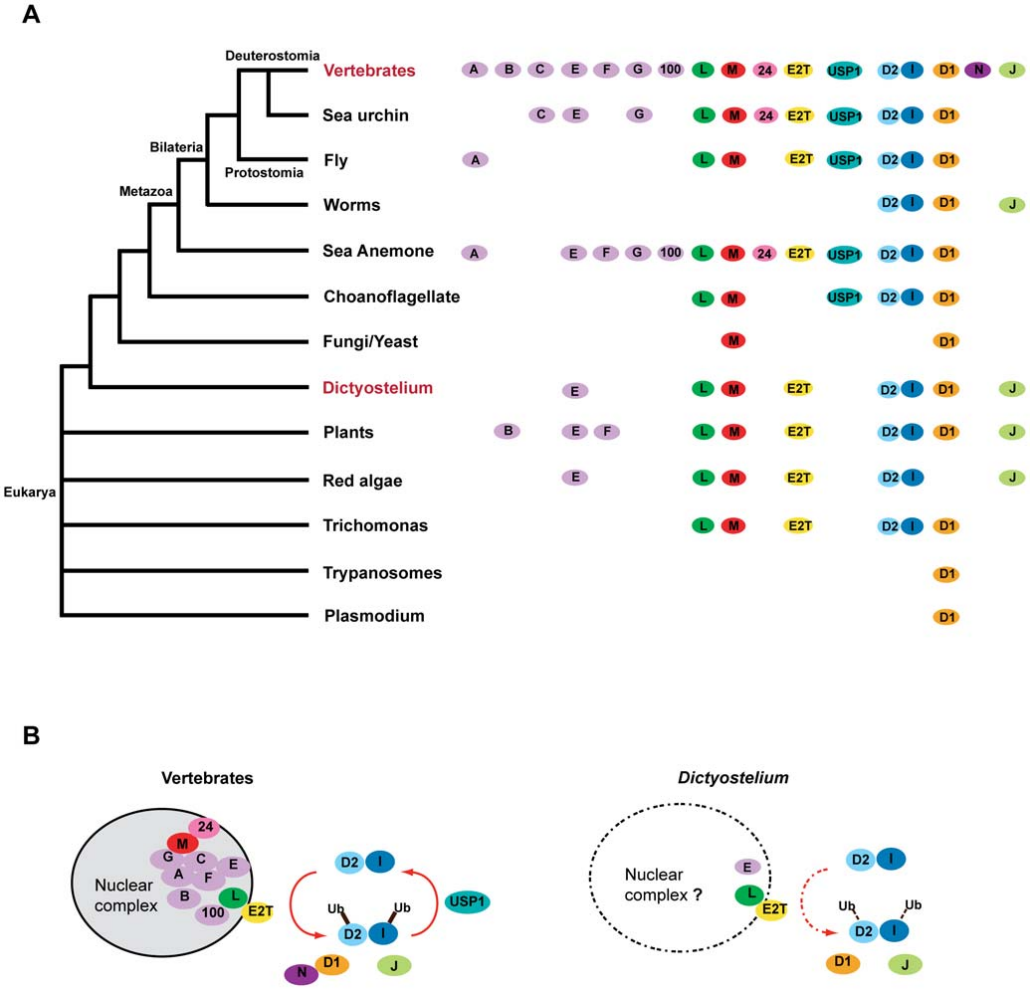
Simplified Fanconi Anaemia crosslink repair pathway in lower eukaryotes. (A) An outline of the conserved Fanconi Anaemia crosslink repair genes in eukaryotic lineages. Most of the FA genes are part of a nuclear core complex. Simpler eukaryotes do not appear to have clear orthologues of many of the core complex genes. Representative species of the lineages are: vertebrate (Homo sapiens, *Mus musculus*, *Gallus gallus*, *Xenopus laevis*, *Danio rerio*), echinoderms (*Strongylocentrotus purpuratus*), insects (Drosophila melanogaster, Anopheles gambiae), worms (*Caenohabditis elegans*), cnidaria (*Nematostella vectensis*), choanoflagellates (*Monosiga brevicollis*), fungi (*Ustilago maydis*), *Dictyostelium discoideum*, plants (*Arabidopsis thaliana*), rhodophytes (*Cyanidioschyzon merolae*), parabasilids (*Trichomonas vaginalis*), euglenozoa (*Trypanosoma brucei*, *Trypanosoma cruzi*, Leishmania major), alveolates (*Plasmodium falciparum*). (B) A schematic representation of the vertebrate FA pathway alongside a potentially much simplified FA pathway that operates in *Dictyostelium*. A = FancA, B = FancB, C = FancC, E = FancE, F = FancF, G = FancG, 100 = FAAP100, L = FancL, M = FancM, 24 = FAAP24, E2T = Ube2T, D2 = FancD2, I = FancI, D1 = FancD1/BRCA2, N = FancN, J = FancJ.

The Fanconi anaemia (FA) genes are a particularly important class of DNA crosslink repair genes in vertebrates. Their inactivation in humans leads to Fanconi anaemia – an illness that leads to developmental defects, stem cell attrition and cancer predisposition [5],[29],[30]. There are 13 known FA genes in humans. Most of them (FANCA, B, C, E, F, G, L, M, FAAP100 and FAAP24) assemble into a nuclear complex – hitherto referred to as the FA core complex. This complex interacts with the E2 enzyme Ube2t [31],[32], and monoubiquitinates two other FA proteins FANCD2 and FANCI. Both proteins form a complex and co-localise at sites of DNA damage with FANCD1 (BRCA2), FANCN (PALB2) and the FANCJ helicase [30]. All the FA proteins are highly conserved in vertebrates. As a first step to dissect crosslink repair in *Dictyostelium* we delineated the pattern and depth of their conservation in all eukaryotes. A clear picture emerges from this analysis (Figure 1A): a minimal FA pathway consists of FANCD2 (FncD2), FANCI (FncI), FANCL (FncL), FANCM (FncM), FANCJ (FncJ), Ube2T (Ube2T) and FancD1/BRCA2 (FncD1); the later appears to have evolved in the ancestral eukaryote. Additional components, including most of the FA core complex proteins, evolved later in the ancestral metazoan. With respect to *Dictyostelium*, this analysis suggests a simplified FA pathway may operate in this organism (Figure 1B).

**Figure 2:**
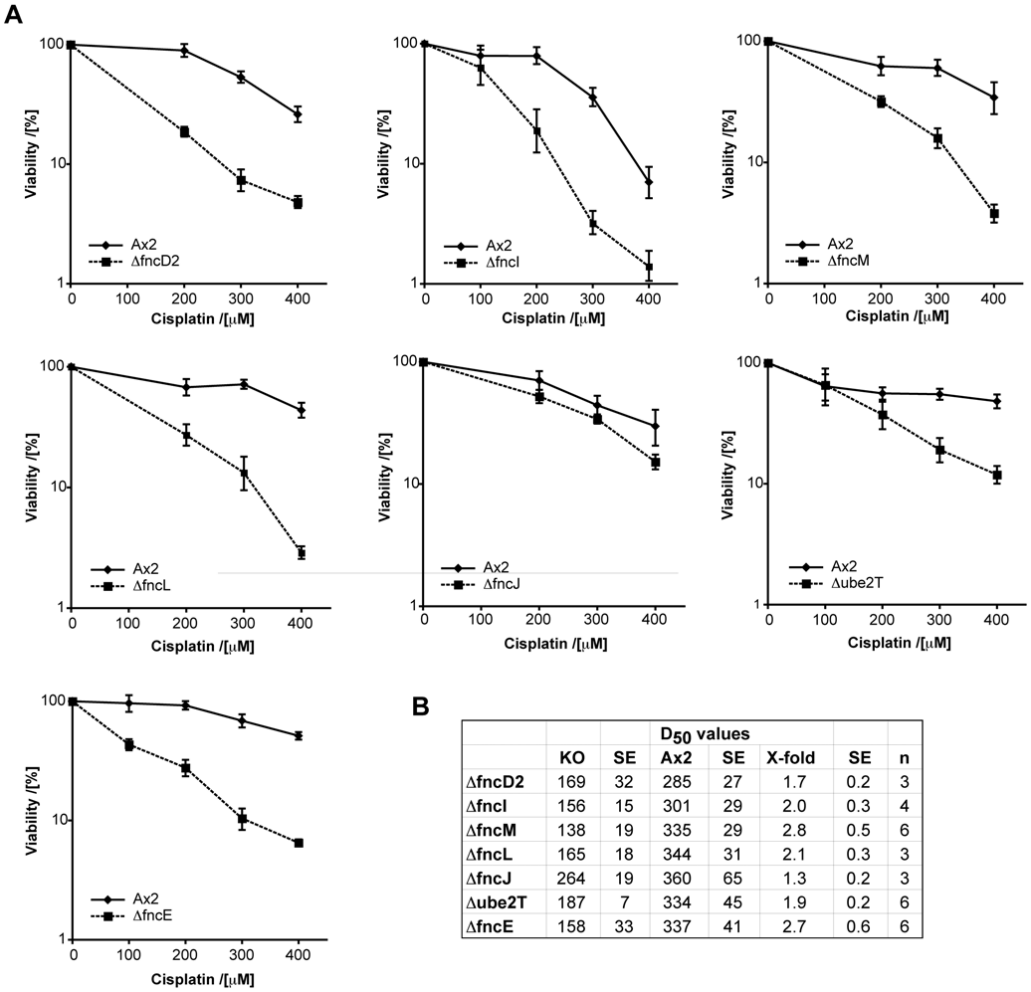
Disruption of the FA genes in *Dictyostelium* leads to sensitivity to the crosslinking agent cisplatin. (A) The genetic loci for f*ncD2*, *fncL*, *fncI*, *fncJ*, *fncM*, and *ube2T* were disrupted and deletion strains were confirmed by Southern analysis (Figure S1, S2, and S3). Viability was estimated by colony survival after exposure to a dose titration of cisplatin for 1 hour prior to plating on bacterial agar lawns. Each survival curve consists of a triplicate experimental data set run in parallel with the wild type (Ax2) control. (B) Table of D_50_ toxicity values for all deletion strains. X-fold represents the difference in the D_50_ value of the relevant strain relative to the wild type control. KO: deletion strain, SE: standard error, n: number of independent experiments carried out.

Next, we proceeded to establish a functional role for the ‘minimal’ FA pathway in *Dictyostelium*. We bioinformatically identified the genomic loci of the *Dictyostelium* FA genes and using these information generated knockouts of orthologues of *FANCD2*, *I*, *L*, *M*, *J* and *Ube2t* (Figures S1, S2, and S3, and Table S1). To study the response to DNA crosslinks, the various *Dictyostelium* strains were exposed to cisplatin. After one hour exposure to a range of doses, the amoebae were diluted, plated out onto bacterial lawns and allowed to grow for 4 days. Surviving amoebae form distinct plaques on the bacterially coated agar plates, each of which represents a colony arisen from a single cell. The number of plaques was counted and survival was expressed as a percentage of plaques formed by mock-treated cells. This assay is very much like the standard colony survival assay used in toxicity studies with vertebrate cell lines. The data in Figure 2 shows that most of the FA knockout strains show a moderate sensitivity to cisplatin. A notable exception is the *fncJ* knockout, which does not seem to be sensitive. Also of note is the dose of mutagen required to compromise wild type cells, which is in the range of 300 µM. This is a very large dose considering that human and chicken cells show sensitivities in the 1–40 nM range. This difference becomes even more striking when comparing the chicken *fancL* knockout, which has a D**_50_** value of 5 nM (8 fold more sensitive compared to wild type), to its *Dictyostelium* counterpart, which has a D_50_ value of 165 µM (2 fold more sensitive than wild type). We can conclude that, firstly, *Dictyostelium* is much more resistant to cisplatin than vertebrate cells. Secondly, the identifiable FA genes are functionally required for this resistance, though unlike in vertebrates their overall contribution is much less marked.

### An FncL protein complex monoubiquitinates FncD2 in *Dictyostelium*

**Figure 3:**
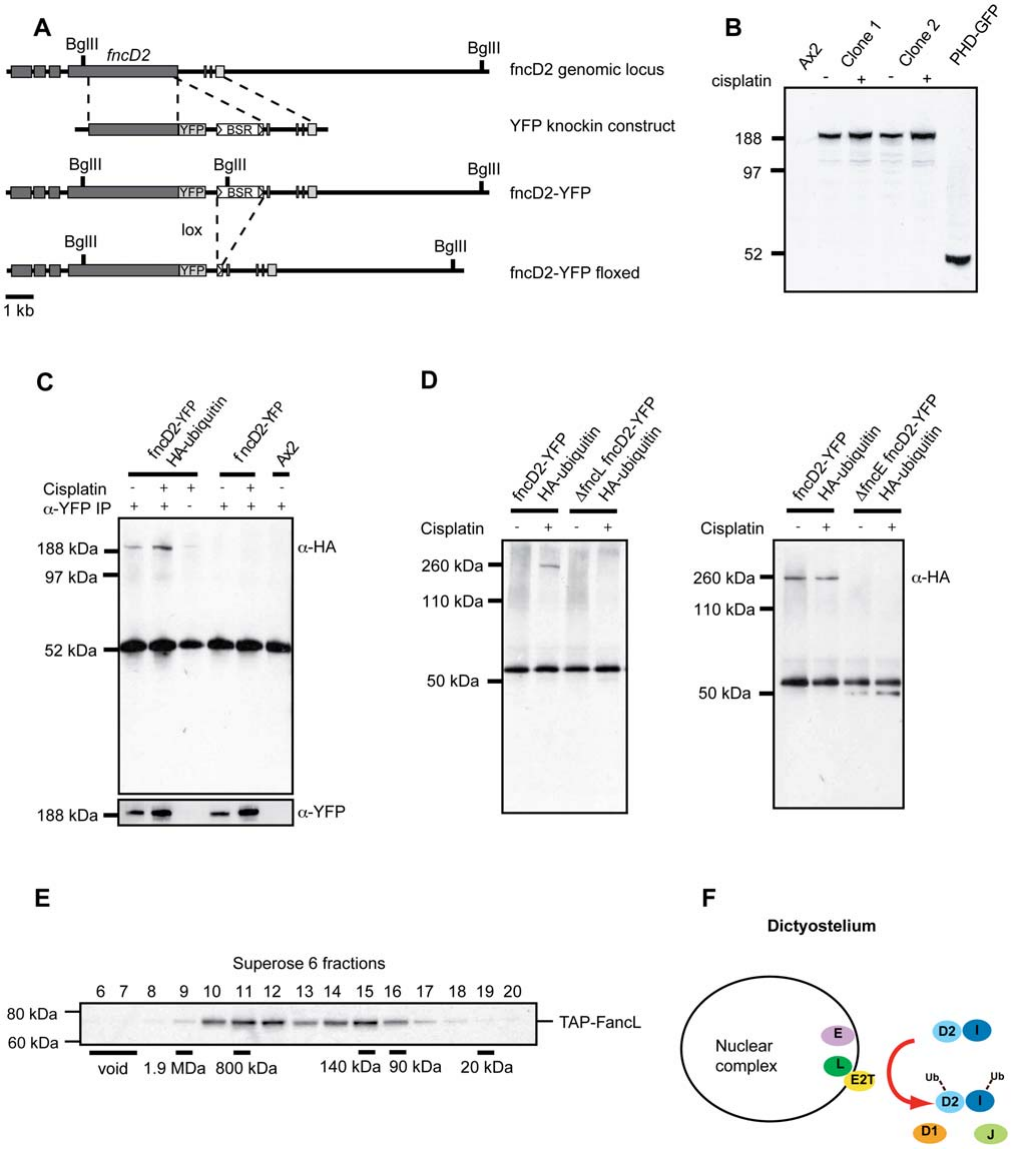
An FncL complex is required for FncD2 monoubiquitination in *Dictyostelium*. (A) Map of the *fncD2* locus (DDB_G0268216) and design of 3′ YFP-knockin strategy. The fncD2 coding region is shown in dark. (B) An anti-YFP Western blot performed on extracts obtained from Ax2 cells, two FncD2-YFP knockin clones, and cells expressing a YFP-tagged PHD finger protein (positive control). FncD2-YFP is expressed from its endogenous locus and migrates only as a single band. (C) To detect monoubiquitinated FancD2-YFP, HA-ubiquitin was constitutively expressed in FancD2-YFP knockin cells. Whole cell lysates prepared from mock treated (−) cells or cells exposed to 400 mM cisplatin (+) for 1 hour were immunoprecipitated with an anti-YFP antibody and then Western blotted with anti-HA (top) or anti-YFP (bottom) antibodies respectively. DNA damage induces FncD2 expression and this correlates with increased monoubiquitination. (D) Disruption of *fncL* and *fncE* in the fncD2-YFP+HA–ubiquitin strain results in undetectable FncD2 monoubiquitination. (E) Whole cell extract from in situ tagged TAP-FncL cells was subjected to size exclusion chromatography. Fractions were collected and Western blotted with an anti-TAP antibody to detect the tagged FncL protein. TAP-FncL fractionates in large molecular mass peaks. (F) A schematic diagram summarising the FA crosslink repair pathway in *Dictyostelium*.

The monoubiquitination of FANCD2 is a key biochemical step in the FA pathway. In vertebrates this step requires the complete FA core complex, with FANCL and Ube2t forming the catalytic core of this reaction [33]. Studies in at least two non-vertebrate model organisms (flies and worms) confirm that FANCD2 monoubiquitination is conserved [16],[17]. Both these organisms appear to have lost many core complex genes, once again raising the possibility of a minimal FA pathway operating in simpler organisms. *Dictyostelium* provides a unique opportunity to test if this is true since it lacks obvious orthologues of so many FA genes. Our first step was to establish whether FncD2 is monoubiquitinated and then to determine the genetic requirements for this. To facilitate detection of endogenous FncD2 we developed a *FncD2* reporter strain where a YFP-tag was knocked in frame after the penultimate codon in the last exon of this gene (Figure 3A). Western blot analysis (Figure 3B) and cisplatin survival data (Figure S5) confirm that this strain expresses functional FANCD2-YFP and is not sensitive to cisplatin. In order to detect monoubiquitinated FncD2 we expressed HA-tagged ubiquitin in the FncD2-YFP strain.

Cell extracts prepared from cisplatin or mock-treated cells were immunoprecipitated with an anti-YFP antibody and Western blotted for the HA-tag. A single DNA damage-inducible band, which corresponded to the size of FncD2-YFP was detected (Figure 3C). We then knocked out *fncL* in this strain and found that monoubiquitinated FncD2-YFP was no longer detectable (Figure 3D). Our next step was to determine if *FncL* acted alone or as part of a complex. Our bioinformatics analysis presented in Figure 1 revealed a possible FANCE orthologue - FncE (Figure S4). FANCE is an essential component of the vertebrate FA nuclear complex. We deleted this gene and found that the resultant Δ*fncE* strain was moderately sensitive to cisplatin (Figure 2) and that monoubiquitinated FncD2-YFP was no longer detectable (Figure 3D). Finally, we needed to determine if any one of these FA core complex proteins exists in a complex. To assay for this, we generated a strain that expresses N-terminal TAP-tagged FncL (Figure S5). Whole cell extract from this strain was subjected to size exclusion chromatography and fractions were blotted for TAP-FncL. The data in Figure 3E clearly show that TAP-FncL is present in two large molecular size peaks of approximately 800 kDa and 140 kDa respectively (Figure 3E). In summary, this data shows that FncL and FncE are required for FncD2 monoubiquitination (Figure 3F). Since FncL appears to reside in a protein complex it is unclear whether a truly ‘minimal’ FA pathway operates in this simple organism.

### Rev3 functions with FncD2 to repair crosslinks

**Figure 4:**
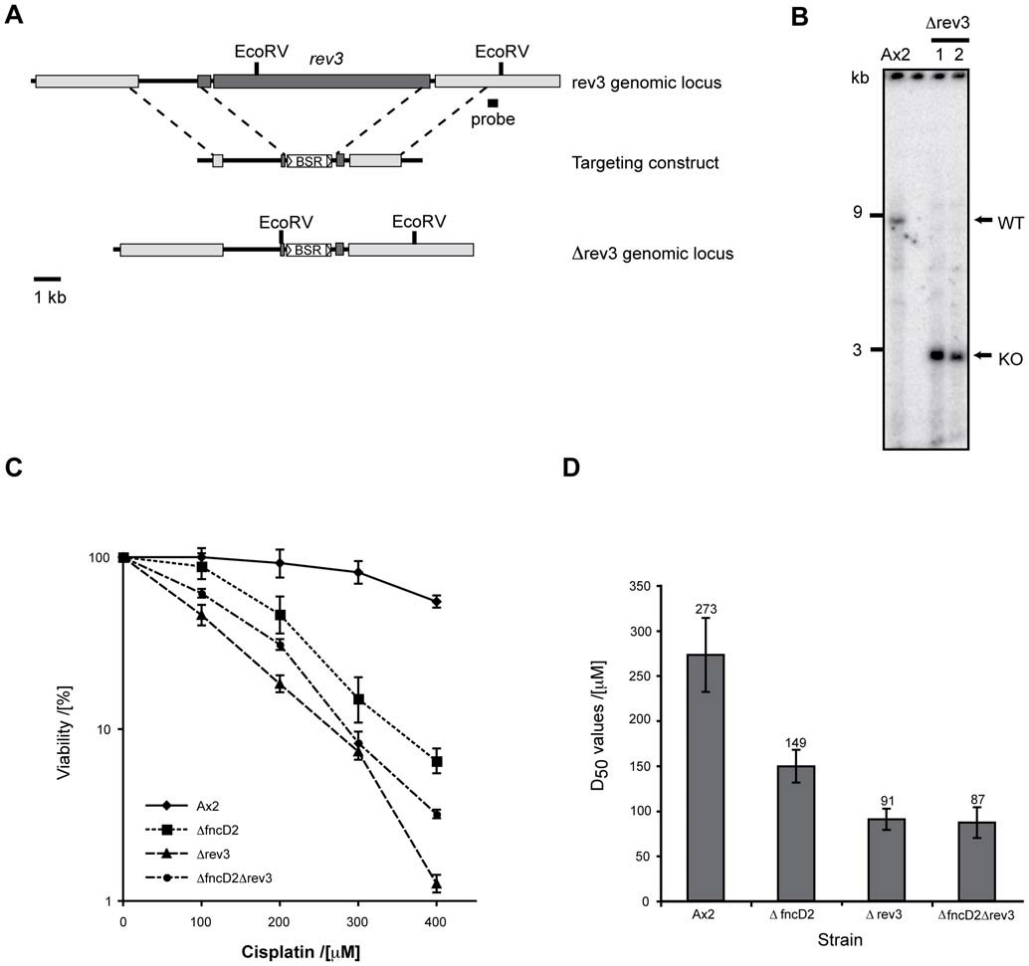
The TLS polymerase Rev3 and FncD2 function in the same process to repair crosslinks in *Dictyostelium*. (A) Map of the intact and disrupted *rev3* locus (DDB_G0271608). r*ev3* exons (dark) are largely deleted by the used knockout strategy. (B) Southern blot of Ax2 cells and two Δrev3 cell lines. Genomic DNA was digested with *EcoR*V and probed with the DNA region marked in (A). The wildtype band is 8.6 kb, which is converted into 2.8 kb band if gene targeting was successful. (C) Colony survival of wild type, Δ*fncD2*, Δ*rev3*, and Δ*rev3*Δ*fncD2* double knockout cells following exposure to cisplatin. Knocking out *rev3* in a Δ*fncD2* knockout background has no synergistic impact on sensitivity to crosslinks. The survival curve consists of a triplicate experimental data set run in parallel with the wild type (Ax2) control. (D) Graphical representation of the D_50_ values of the strains in (C), calculated from five independent colony survival experiments.

**Figure 5:**
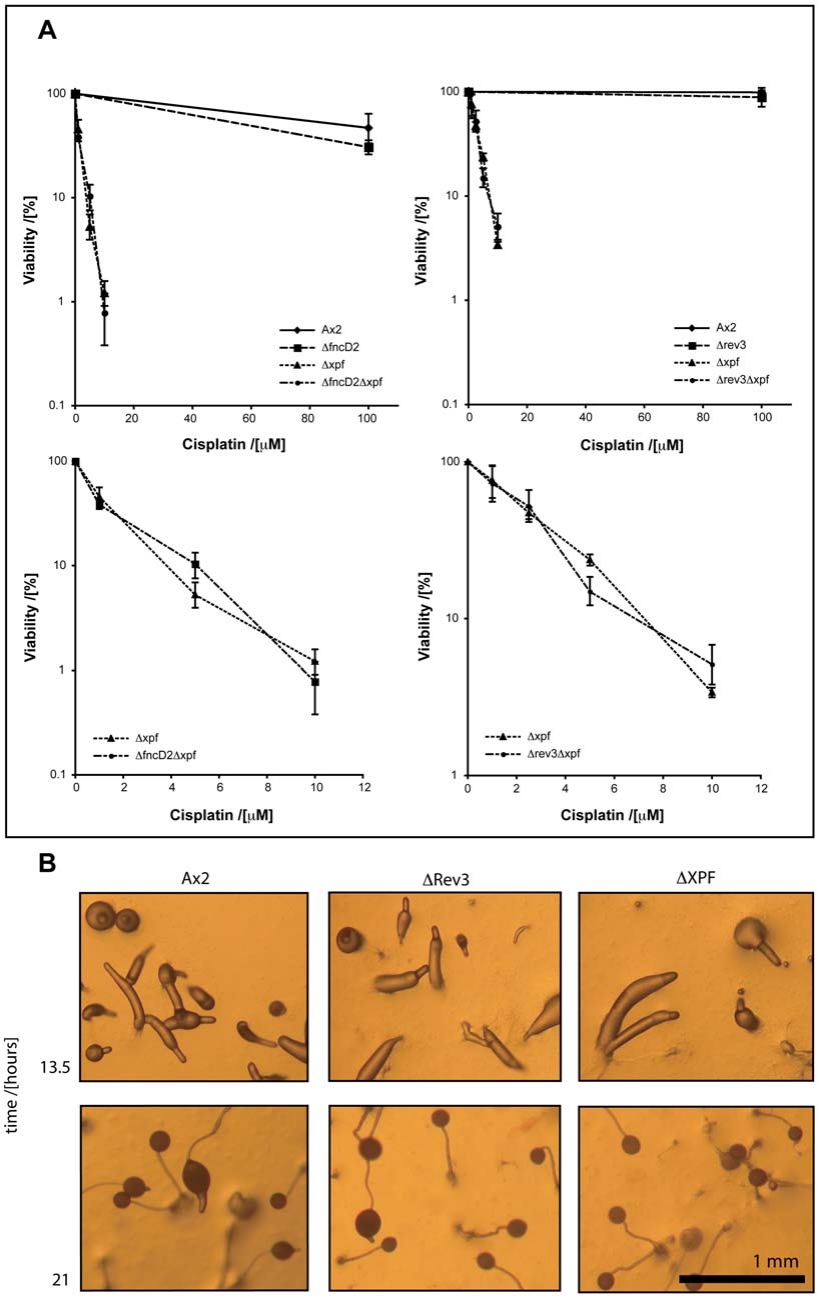
Xpf functions largely independently of either Rev3 or FncD2 in crosslink repair. (A) The Δ*fncD2*Δ*xpf* and the Δ*rev3*Δ*xpf* double knockout strains are as sensitive to cisplatin as the single Δ*xpf* knockout (assayed by colony survival). Results shown are from a single experiment, which is representative of at least three independent experiments. Error bars indicate variation between triplicate plating. The kill curves are shown with a concentration range from 0–100 µM (top of the panel) and also 0–10 µM (bottom). (B) Development is not impaired in Δ*xpf* and Δ*rev3* strains.

A recent study surveyed the relative sensitivity of a large number of DNA repair mutants generated in the isogenic chicken cell line DT40 [34]. This comparison revealed that the most sensitive mutants are those that lack the translesion polymerases Rev1 and Rev3, followed closely by mutants that lack the FA genes. Analysis of double mutants within these two groups of genes in DT40 shows that they participate in a common process to repair crosslinks [11]. These observations prompted us to establish the role of TLS in *Dictyostelium* crosslink repair. The *Dictyostelium* genome appears to contain a smaller complement of TLS enzymes than vertebrates. However a Rev3 orthologue (*rev3*) was easy to identify. We disrupted the *rev3* locus, the ensuing Δ*rev3* strain (Figure 4A and 4B) was viable, grew normally in culture and showed normal development (Figure 5B). We then tested the Δ*rev3* for sensitivity to cisplatin and were surprised to see that it was only moderately sensitive to this agent (3 fold over WT) (Figure 4C). We then disrupted *fncD2* in this strain to test the genetic interaction between these two crosslink repair genes. *fncD2* deficiency makes no additional or synergistic impact in the Δ*rev3* strain (Figure 4C and 4D), indicating that both genes function in a common process to repair crosslinks. However, notably the Δ*rev3* strain (like the FA mutants) was not strongly sensitive to crosslinks, once again contrasting with the corresponding sensitivities seen for this mutant in vertebrate cells.

### The nucleotide excision repair nuclease subcomponent XPF is essential for crosslink repair

**Figure 6:**
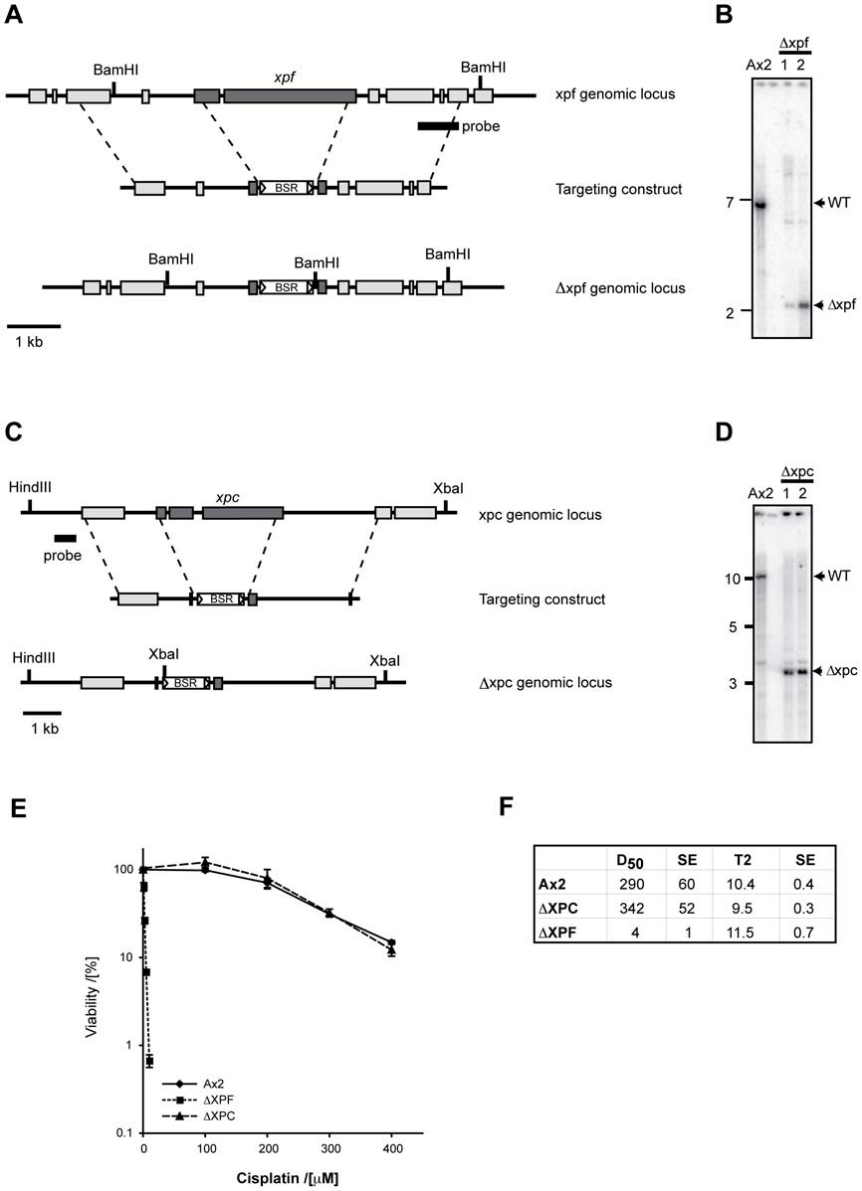
Disruption of the NER nuclease subcomponent *xpf* but not *xpc* results in profound sensitivity to cisplatin in *Dictyostelium*. (A) Map of the intact and disrupted *xpf* locus (DDB_G0284419). *xpf* exons (dark) are largely deleted by this strategy. (B) Southern blot of Ax2 cells and two *xpf* knockout clones. Genomic DNA was digested with *BamH*I and probed with the DNA region marked on the map in (A). The WT band is 6.6 kb, which is converted into a 2.5 kb band with successful gene targeting. (C) Map of the intact and disrupted *xpc* locus (DDB_G0292296). *xpc* exons (dark) are largely deleted by this strategy. (D) Southern blot of Ax2 cells and two *xpc* knockout clones. Genomic DNA was digested with *Xba*I and probed with the DNA region marked on the map in (C). The WT band is 10.6 kb. With successful gene targeting the band is converted into a 3.3 kb band. (E) Colony survival of wild type, Δ*xpc*, and Δ*xpf* cells after exposure to cisplatin. Knocking out *xpf* results in a profound sensitivity to cisplatin. (F) A table showing the doubling time (T2) and D_50_ values for Δ*xpf* and Δ*xpc* strains. SE: standard error.

The fact that *Dictyostelium* mutants of FA and TLS genes are only moderately sensitive to cisplatin surprised us. This organism may be resistant to cisplatin because of reduced bioavailability of the drug (reduced uptake/enhanced breakdown). In addition, it is noteworthy that the *Dictyostelium* genome is very AT-biased [20]. Since cisplatin crosslinks form at mainly GC sequences, this could mean that very few lesions are produced. Alternatively, crosslink repair may be carried out by another process that does not use the FA and TLS genes studied here. One obvious pathway would be HR. However, to date we and others have not been able to knockout core genes in this pathway [35],[36]. Perhaps this could be due to an essential role for HR in cell viability. Another candidate group of genes are those involved in nucleotide excision repair. Vertebrate cell lines lacking NER show differential requirements for crosslink repair. Certain genes like *xpa* and *xpc* play at best only a minor role [9],[37], whilst the nuclease subcomponent XPF appears to be very important. Indeed, all models of crosslink repair invoke the action of a nuclease in cutting on either side of the crosslink, a step referred to as unhooking. In addition to XPF/ERCC1, the Mus81/EME1 nuclease complex is also believed to be important in vertebrate crosslink repair [12],[38],[39]. We therefore set out to disrupt XPF (*xpf*), XPC (*xpc*) and Mus81 (*mus81*). The respective orthologues were identified, their loci mapped and disrupted (Figure 6A–6D and Figure S6A and S6B). All three mutant cell lines were then tested for sensitivity to cisplatin. The Δ*xpc* and Δ*mus81* strains were not sensitive (Figure 6E and Figure S6C), in contrast to the Δ*xpf* mutant, which was extremely sensitive to cisplatin. The D**_50_** values reflect this with Δ*xpf* giving a value of 4 µM, in contrast with Δ*xpc* (342 µM) and wild type (290 µM) (Figure 6F). In summary, the excision repair nuclease subcomponent *xpf* is essential for repairing crosslinks in *Dictyostelium*. This activity is not due to the role of this gene in global NER since the *xpc* mutant is not at all sensitised to cisplatin.

### *xpf* functions largely independently of *rev3* and *fncD2*

The current models for crosslink repair all involve a nuclease(s) carrying out the excision step. Our discovery of an essential role played by *xpf* and not *mus81* in crosslink repair makes *xpf* a very good candidate component for the nuclease implicated in such a step. In a *Xenopus* cell free system, a crosslinked plasmid was repaired in a replication process that involves excision and TLS [40]. This important study therefore raises the question regarding the identity of the nuclease(s) involved in this excision step. A genetic test to determine if XPF might be involved here is to generate a Δ*xpf*Δ*rev3* strain and to establish genetic epistasis between these genes. If the double mutant is as sensitive as the single Δ*xpf* strain then this indicates that *xpf* functions with *rev3* in crosslink repair. This is indeed what we see since disruption of *rev3* does not impact further on the sensitivity to cisplatin in the Δ*xpf* strain (Figure 5A). In addition, we also demonstrated that *xpf* is epistatic with respect to *fncD2* (Figure 5A). The single Δ*xpf* mutant is 20–30 fold more sensitive than either Δ*rev3* or Δ*fncD2* strains, respectively, which indicates that most crosslink repair requires *xpf* but not *rev3* or *fncD2*. Finally there is considerable evidence for the role of XPF in HR repair [41]–[43]. To test whether HR repair is compromised in Δ*xpf* we analysed gene targeting efficiency into two independent loci. The data in Table 1 clearly shows that homologous gene targeting is compromised to varying degrees in both loci analysed in the Δ*xpf* strain compared to wild type AX2.

**Table 1:** Measured gene-targeting frequencies into two loci in WT and Δ*xpf* cells.

Strain	Gene Targeted	
	DDB_G0293840	DDB_G0267916
WT	24% (12/50)	80% (131/170)
Δxpf	0% (0/25)	2% (12/518)

The table depicts the gene-targeting efficiencies into two independent genetic loci of WT and Δ*xpf* cells. For each locus multiple transfections were performed and drug-resistant clones were analysed by PCR for proper targeting. The number of gene-targeting events is shown alongside the total number of drug resistant clones. Gene-targeting efficiency is expressed as a percentage value of the total number of clones.

## Discussion

The studies presented in this paper establish the genetic requirements for crosslink repair in *Dictyostelium*. This simple unicellular genotoxin-resistant eukaryote shares the important groups of crosslink repair genes that function in humans. In contrast to vertebrates, the FA proteins and TLS enzymes appear to play only a minor role in repairing crosslinks. However, the most striking discovery reported here is that the nucleotide excision repair gene *xpf* is essential for crosslink repair in *Dictyostelium*.

So far our analysis has confirmed that at least two known proteins that are crucial for the function of the FA core complex, FANCE and FANCL, are conserved in this organism. FANCE links the FA complex to its main substrate FANCD2 [44] and FANCL is the E3 subunit in the complex [45]. An unresolved question is whether *Dictyostelium* has a truly simplified FA pathway. Certainly the FncL protein exists in a high molecular mass protein complex. Such a complex may consist only of FncL and FncE. Another possibility is that there are other proteins in this complex. Such proteins could be other FA core complex orthologues that have simply evaded detection by bioinformatics as they may have diverged at the amino acid sequence level but not at a structural level. Alternatively, both FncL and FncE could be embedded in a complex consisting of new proteins or into a known surrogate multiprotein E3 ligase complex. Purification and identification of components of the FncL complex should address these possibilities.

It has long been appreciated that *Dictyostelium* is an unusually DNA damage-resistant organism. The work presented here further illustrates this. Conceivably factors such as reduced bioavailability of cisplatin and the number of crosslinks introduced into the genome may contribute towards this resistance. However, the profound cisplatin sensitivity as a result of *xpf* inactivation clearly showed that a sufficient number of crosslinks are formed to cause lethal damage. We were surprised that the FA and the TLS proteins seem to only contribute a minor activity towards this crosslink resistance. This finding contrasts with what has been observed with vertebrate cells, where both groups of genes are crucial for tolerance to DNA crosslinking agents. The profound sensitivity of the xpf-deficient strain raises the question how xpf contributes to tolerance to DNA crosslinking agents repair. The genetic interactions between *xpf*, *fncD2* and *rev3* show that there is a minor repair process involving all three genes, but the dominant mechanism of xpf-dependent crosslink repair remains to be determined.

**Figure 7:**
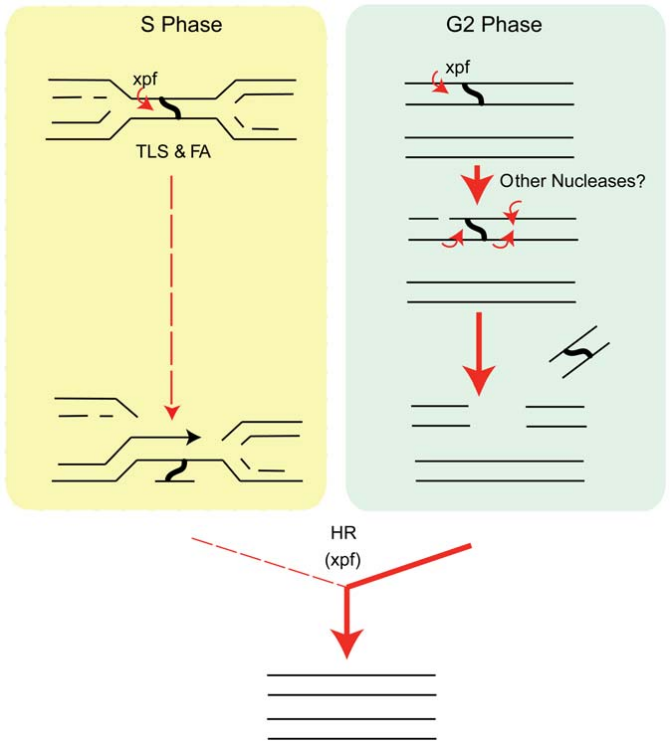
Model for an Xpf-dependent crosslink repair process in *Dictyostelium*. Model outlining a potential mechanism of crosslink repair that may operate in *Dictyostelium*. A small proportion of repair is channelled through a combined FA, TLS, and HR route stimulated by replication fork stalling. However, as *Dictyostelium* spends most of its time in the G2 phase of the cell cycle it is likely that a crosslink may form at only one site on a particular chromosome. A simple mechanism may operate whereby Xpf/Ercc1 together with other nucleases may cut out the crosslinked section creating a double strand break. HR using the sister chromatid as a template might then complete this repair process. It is also possible that *Xpf* plays an additional role in the HR step.

During vegetative growth, *Dictyostelium* displays a skewed cell cycle distribution. The vast majority of cells are found in the G2 phase of the cell cycle [46],[47]. The G1 phase appears to be very short. This, in terms of DNA content, means that most of the vegetative cells possess a duplicated genome. Upon exposure to cisplatin, it is very likely that lesions form at only one copy per site. Under such conditions, the most straightforward means of repair would be to excise the crosslink creating a double strand break. The undamaged copy could then be used as the template for HR-mediated double strand break repair (Figure 7). Such a model predicts the requirement for HR genes in crosslink repair, a proposition that is currently difficult to address, since we and others thus far been unable to disrupt HR genes in *Dictyostelium* [35],[36]. In addition, this model of crosslink repair may require additional nucleases to not only to create but also to process DNA double strand breaks, remove flaps or resolve secondary structures. All these activities would be quite distinct from the unhooking step itself.

Considerable work in both yeast and vertebrates point to a critical role for XPF and its orthologues in homologous recombination repair [42],[48]. ES cells and yeast knockouts appear to be defective at homologous gene targeting though it is important to appreciate that this is not always a consistent feature [41],[43]. Recombinant XPF/ERCC1 also function in processing recombination intermediates as well as synthetic replication forks [49]. Indeed we also find a defect in gene targeting in the Δ*xpf* strain indicating that like in other organisms, Xpf does play a role in HR in *Dictyostelium*. It is therefore possible that it is the HR functions of Xpf that determines why it is so crucial for the tolerance of crosslinks in *Dictyostelium*. Finally, it is noteworthy that there are many organisms that share resistance to DNA damaging agents with *Dictyostelium*. The dependence of an excision nuclease-based repair mechanism may be responsible for such resistance. Such a mechanism may not just be limited to such organisms but also to human cancers which develop resistance to cisplatin [50]. Induction of such an excision repair pathway may account for the acquired resistance to chemical crosslinking agents. Future work will aim to address the genetic requirements and elucidate the mechanism of the xpf-dependent crosslink repair pathway.

## Materials and Methods

### Strain generation

All targeting constructs were generated using pLPBLP as the backbone [51]. 5′ and 3′ homology arms were generated by PCR amplification from Ax2 genomic DNA using *Pwo* polymerase and inserted into the plasmid on either side of the blasticidin-resistance cassette (bsr). The HA-ubiquitin overexpression construct was generated using pDXA-3C as backbone [52].The wild-type strain and the parent of all strains generated in this study was the Kay laboratory version of Ax2. Transformants were created by electroporation (Genepulser Xcell Bio-Rad) of 17.5 µg of the targeting cassette or 25 µg of the overexpression plasmid. Potential homologous recombinants were selected for blasticidin resistance (10 µg/ml) at limiting dilution in 96-well plates, whereas overexpression lines were selected as a pool of transformants in the presence of 10 µg/ml G418. After approximately 10 days, the content of positive wells were cloned out onto SM agar plates in association with *K. aerogenes*. Colonies were picked and analysed by PCR. Genomic DNA was prepared from approximately 3×10^6^ cells using the GenElute Mammalian Genomic DNA Miniprep Kit (Sigma) according to manufacturer's protocol. Two screening primers were designed per strain, one placed just upstream of the 5′ homology arm (primer X) and another just downstream of the 3′ homology arm (primer Y) in the genomic sequence. Each of the two primers was paired with a primer of the appropriate sense that bound within the bsr cassette (BSR1B and BSR2B). The generation of a product by primer X and BSR1B, and primer Y and BSR2B indicated that the bsr cassette had integrated into the correct genomic locus.

BSR1B - 5′ – CATTGTAATCTTCTCTGTCGCTACTTCTAC – 3′

BSR2B - 5′ - GTGTAGGGAGTTGATTTCAGACTATGCACC – 3′

All disrupted strains were confirmed by Southern analyses according to standard protocol. Genomic DNA was extracted using a method adapted from a universal, rapid high-salt extraction protocol [53]. When further genetic manipulation (either gene disruption or *in situ* tagging) of a knockout strain was required, the *bsr* cassette was removed from the parental strain by transfection with pDEX-NLS-Cre [51] and selecting for G418 resistance (10 µg/ml). After approximately 10 days of selection, resistant cells were cloned out onto SM-agar plates in the presence of *K. aerogenes* and tested for blasticidin (10 µg/ml) and G418 (10 µg/ml) sensitivity in axenic media.

### Cell culture

All strains were routinely grown at 22°C in axenic medium [19] supplemented with vitamins (0.1 mg/l B12, 0.02 mg/l Biotin, 0.2 mg/l Riboflavin) in the presence of tetracycline (10 µg/ml) and streptomycin (200 µg/ml), either in tissue culture plates or in conical flasks shaken at 180 rpm (shaken suspension). Strains can also be cultured in association with *Klebsiella aerogenes* on SM agar plates. Strains carrying pDXA-3C-based *neo* ^R^-expressing plasmids were grown in axenic medium supplemented with G418 (10 µg/ml).

### *Dictyostelium* development

Axenically grown cells in log phase (2–5×10^6^ cells/ml) were harvested by centrifugation (200 *g*, 2 minutes) and washed twice with KK2 buffer (16.5 mM KH_2_PO_4_, 3.9 mM K_2_HPO_4_, pH 6.1). Cells were resuspended in KK2 plus 0.1 mM CaCl_2_ to 2.5×10^7^ cells/ml and 4 ml (10^8^ cells) were plated per agar plate (1.5×10^6^ cells/cm^2^) in duplicate. Cells were allowed to settle on the agar for 15 minutes before the buffer was aspirated. Plates were then incubated in a moist box at 22°C with light. Photographs were taken with a Nikon Coolpix 4500 camera mounted on a Wild M10 microscope at the indicated time points in development.

### Colony survival assay

Cells in logarithmic growth phase (2–6×10^6^ cells/ml) were harvested, resuspended at 1×10^6^ cells/ml in Pt buffer (3 mM NaCl, 1 mM NaPO_4_, pH 6.5) and treated with cisplatin (Sigma) or mock-treated for 1 hour at 22°C in shaken suspension in the dark. The cisplatin solution was prepared in the dark immediately prior to use by dissolving in Pt buffer to a concentration of 1 mg/ml (3.3 mM). After treatment, cells were serially diluted in KK2 buffer and 50 µl of two dilutions shown to contain approximately 50 viable cells in preliminary experiments were plated in triplicate on SM agar plates with 400 µl of two-day old *K. aerogenes* culture. The plates were incubated at room temperature and the number of plaques per plate was scored 4 days after plating. An average was taken between the triplicate plates. Viability was calculated as a percentage of the estimated number of cells plated, which was then normalised against that of the mock-treated culture.

### Immunoprecipitation

Typically, 10^8^ cells were harvested by centrifugation and washed twice with 1 ml KK2 buffer. The cell pellet was resuspended in 500 µl NETN lysis buffer (20 mM Tris-HCl pH 8.0, 150 mM NaCl, 1 mM EDTA, 1 mM DTT, 0.5% NP40, 10% glycerol, 1× Protease Inhibitor Cocktail [Roche], 5 mM NEM [Sigma]). The lysate was drawn through a 25G needle four times to ensure complete lysis of the cells and to shear the genomic DNA. The resulting whole cell extract was cleared by centrifugation and the protein concentration was determined by Bradford assay. Protein concentrations across all samples were equalised and the total extract volume was made up to 540 µl with NETN lysis buffer. 1 µl of rabbit polyclonal antibody to GFP (Abcam ab6556) was added and mixed by rotation during an 1 hour incubation at 4°C. 200 µl of 50 mg/ml freshly prepared protein A-sepharose beads (GE Healthcare) were then added and the samples mixed and incubated as the previous step. The beads were then pelleted by centrifugation, washed four times with 1 ml NETN lysis buffer and finally resuspended in 100 µl 2× SDS loading buffer.

### Western blotting

Protein samples were run on 10% NuPAGE Bis-Tris pre-cast gels (Invitrogen) in 1× MOPS buffer (Invitrogen). The separated proteins were transferred onto nitrocellulose membrane (Millipore). After blocking with 5% milk/PBST, the blot was incubated with the appropriate primary and secondary antibody diluted in PBST (PBS with 0.05% v/v Tween-20) for 1 hour each at room temperature. The following antibodies and dilutions were used: rabbit anti-GFP antibody ab6556 (Abcam; 1∶2000), goat anti-rabbit IgG HRP-conjugated antibody (Southern Biotech; 1∶1000–1∶2000), mouse monoclonal anti-HA (clone 12CA5) HRP-conjugated antibody (Roche; 1∶1000), rabbit anti-TAP antibody (Sigma-Aldrich; 1∶600).

### Size exclusion chromatography

2×10^9^ exponentially growing cells were harvested and washed three times with KK2 buffer before flash-freezing in liquid nitrogen and storing at −80°C until use. The cell pellet was resuspended in 10 ml high salt buffer (50 mM HEPES pH 7.9, 5 mM MgCl_2_, 420 mM NaCl, 0.2 mM EDTA, 25% glycerol, 2 mM DTT, 1× Protease Inhibitor Cocktail [Roche]) on ice. The suspension was taken up in a syringe and forced through a 3 µm Nucleopore filter (Whatmann) and absorbant pad (Millipore) to complete cell lysis, and was subsequently passed through a 26G needle to lyse the nuclei. The resulting lysate was mixed gently at 4°C for 30 minutes to extract nuclear protein and cleared by centrifugation (16,000 *g*, 10 minutes at 4°C). 2 ml whole cell extract was filtered through a 0.2 µm filter and applied to a Superose 6 XK 16/70 column (GE Healthcare) equilibrated with high salt buffer. 4 ml fractions were collected and 25 µl of each fraction was resolved on 10% Bis-Tris polyacrylamide gels and analysed by Western blotting.

### Computational methods

Orthologue searches were done using two publicly available databases – NCBI BLAST Link (BLink) and Kyoto Encyclopaedia of Genes and Genomes (KEGG) Orthology.

NCBI BLink – http://www.ncbi.nlm.nih.gov/sites/entrez

KEGG Orthology – www.genome.jp/kegg/

PSI-BLAST searches were carried out using the NCBI Blastp suite.

http://blast.ncbi.nlm.nih.gov/Blast.cgi

Structure of the *Dictyostelium* FancE orthologue was predicted using Phyre.

http://www.sbg.bio.ic.ac.uk/~phyre/

Sequence alignments were carried out using ClustalW [54] and displayed using JalView (http://www.jalview.org/).

## Supporting Information

Figure S1 Generation and verification of the Δ*fncD2* and Δ*fncI* null strains. (A) Generation and verification of the Δ*fncD2* strain. Schematic representation of the targeting construct used for knocking out *fncD2* (DDB_G0268216) and location of the probes and restriction sites used for Southern blot analysis. This analysis resulted in a 17.5 kb band for WT cells and a 7.9 kb band for Δ*fncD2* strains. (B) Generation and verification of the Δ*fncI* strain. Schematic representation of the targeting construct used for knocking out *fncI* (DDB_G0293476) and location of the probes and restriction sites used for Southern blot analysis. This analysis resulted in a 10.7 kb band for WT cells and a 7.1 kb band for Δ*fncI* strains.
(2.75 MB TIF)

Figure S2 Generation and verification of the Δ*fncM* and Δ*fncL* null strains. (A) Generation and verification of the Δ*fncM* strain. Schematic representation of the targeting construct used for knocking out *fncM* (DDB_G0274841) and location of the probes and restriction sites used for Southern blot analysis. This analysis resulted in an 11.2 kb band for WT cells and a 4.6 kb band for Δ*fncM* strains. (B) Generation and verification of the Δ*fncL* strain. Schematic representation of the targeting construct used for knocking out *fncL* (DDB_G0292744) and location of probes and restriction sites used for Southern blot analysis. This analysis resulted in a 13.9 kb band for WT cells and a 4.4 kb band for Δ*fncL* strains.
(5.55 MB TIF)

Figure S3 Generation and verification of the Δ*fncJ* and Δ*ube2T* null strains. (A) Generation and verification of the Δ*fncJ* strain. Schematic representation of the targeting construct used for knocking out *fncJ* (DDB_G0286621) and location of the probes and restriction sites used for Southern blot analysis. This analysis resulted in a 9.1 kb band for WT cells and a 4.7 kb band for Δ*fncJ* strains. (B) Generation and verification of the Δ*ube2T* strain. Schematic representation of the targeting construct used for knocking out *ube2T* (DDB_G0291199) and location of the probes and restriction sites used for Southern blot analysis. This analysis resulted in a 6.3 kb band for WT cells and a 7.3 kb band for Δ*ube2T* strains.
(6.15 MB TIF)

Figure S4 Generation and verification of the Δ*fncE* strain. (A) ClustalW alignment of the FncE sequences of *Homo sapiens*, *Mus musculus*, *Gallus gallus*, *Danio rerio*, *Arabidopsis thaliana*, and *Dictyostelium discoideum*. The *Dictyostelium* FncE sequence is highlighted by dashed red lines. (B) Generation and verification of the Δ*fncE* strain. Schematic representation of the targeting construct used for knocking out *fncE* (DDB_G0279669) and location of the probes and restriction sites used for Southern blot analysis. (C) Verification of the Δ*fncE* strain by Southern blot. This analysis resulted in a 4.0 kb band for WT cells and a 5.1 kb band for Δ*fncE* strains.
(3.21 MB TIF)

Figure S5 Generation and verification of the TAP-FncL strain. (A) Schematic representation of the targeting construct used for N-terminal in situ tagging of FncL with TAP. (B) Western blot showing TAP-FncL expression and specific detection of FANCL by the anti TAP antibody. Ax2 lysate was included as a negative control. The lysate of 7.5×10^5^ cells was loaded per lane.
(1.35 MB TIF)

Figure S6 Generation and verification of the Δ*mus81* strain. (A) Schematic representation of the targeting construct used for knocking out *mus81* (DDB_G0276519). (B) Verification of Δ*mus81* clones by Southern blot analysis. This analysis resulted in a 7.1 kb band for WT cells and a 6.0 kb band for Δ*mus81* strains. (C) The Δ*mus81* strain is not sensitive to cisplatin as assayed by colony survival. Results shown are from a single experiment. Error bars indicate variation between triplicate plating.
(1.42 MB TIF)

Figure S7 The FancD2-GFP strain is not sensitive to cisplatin. The FancD2 C-terminal GFP tagged strain does not show sensitivity to cisplatin.
(0.29 MB TIF)

Table S1 Table of all the strains generated and used in this study. The systematic strain name (HMxxxx) is based on the nomenclature used in R. R. Kay's lab. Parental strain, genotype (Δ = deletion), overexpression plasmid present, and drug resistance of each strain are presented.
(0.11 MB DOC)
