# Supplementary figures and images for: Xpf and Not the Fanconi Anaemia Proteins or Rev3 Accounts for the Extreme Resistance to Cisplatin in Dictyostelium discoideum
Source: PLoS Genet. 2009 Sep 18;5(9):e1000645. doi: 10.1371/journal.pgen.1000645 (PMC2730050; doi:10.1371/journal.pgen.1000645)

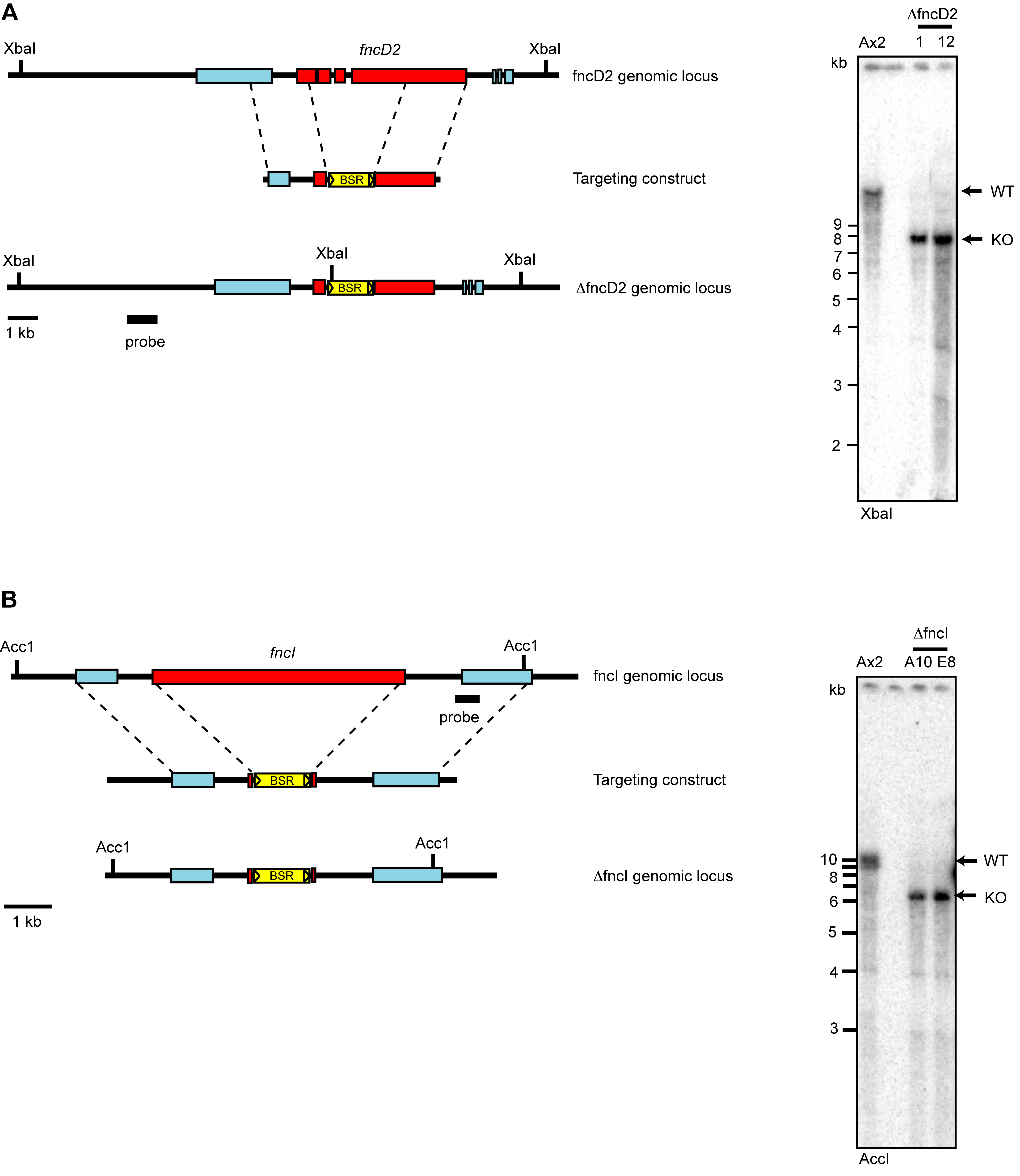

Supplement: Figure S1 — Generation and verification of the ΔfncD2 and ΔfncI null strains. (A) Generation and verification of the ΔfncD2 strain. Schematic representation of the targeting construct used for knocking out fncD2 (DDB_G0268216) and location of the probes and restriction sites used for Southern blot analysis. This analysis resulted in a 17.5 kb band for WT cells and a 7.9 kb band for ΔfncD2 strains. (B) Generation and verification of the ΔfncI strain. Schematic representation of the targeting construct used for knocking out fncI (DDB_G0293476) and location of the probes and restriction sites used for Southern blot analysis. This analysis resulted in a 10.7 kb band for WT cells and a 7.1 kb band for ΔfncI strains. (2.75 MB TIF) [file pgen.1000645.s001.tif]

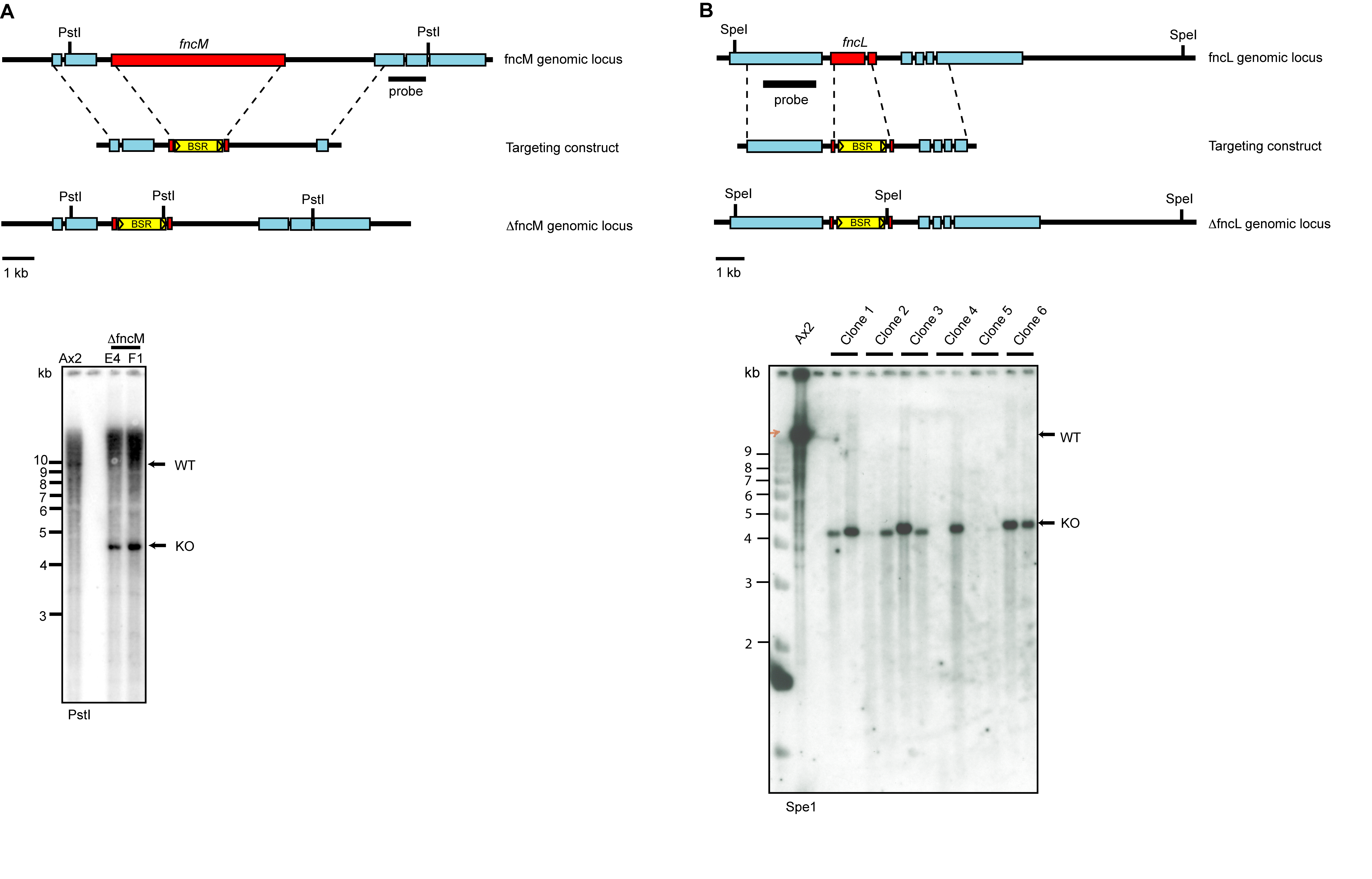

Supplement: Figure S2 — Generation and verification of the ΔfncM and ΔfncL null strains. (A) Generation and verification of the ΔfncM strain. Schematic representation of the targeting construct used for knocking out fncM (DDB_G0274841) and location of the probes and restriction sites used for Southern blot analysis. This analysis resulted in an 11.2 kb band for WT cells and a 4.6 kb band for ΔfncM strains. (B) Generation and verification of the ΔfncL strain. Schematic representation of the targeting construct used for knocking out fncL (DDB_G0292744) and location of probes and restriction sites used for Southern blot analysis. This analysis resulted in a 13.9 kb band for WT cells and a 4.4 kb band for ΔfncL strains. (5.55 MB TIF) [file pgen.1000645.s002.tif]

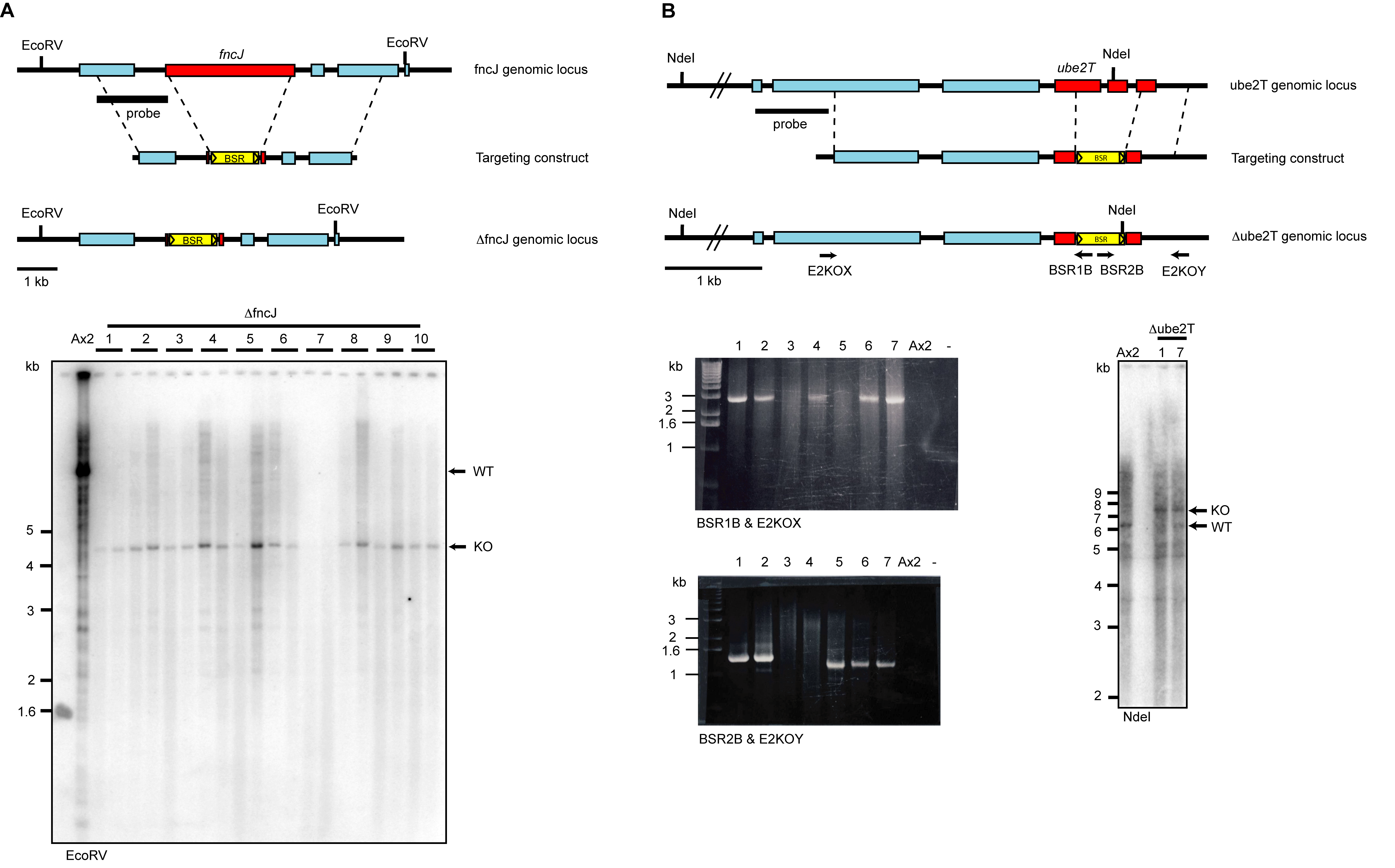

Supplement: Figure S3 — Generation and verification of the ΔfncJ and Δube2T null strains. (A) Generation and verification of the ΔfncJ strain. Schematic representation of the targeting construct used for knocking out fncJ (DDB_G0286621) and location of the probes and restriction sites used for Southern blot analysis. This analysis resulted in a 9.1 kb band for WT cells and a 4.7 kb band for ΔfncJ strains. (B) Generation and verification of the Δube2T strain. Schematic representation of the targeting construct used for knocking out ube2T (DDB_G0291199) and location of the probes and restriction sites used for Southern blot analysis. This analysis resulted in a 6.3 kb band for WT cells and a 7.3 kb band for Δube2T strains. (6.15 MB TIF) [file pgen.1000645.s003.tif]

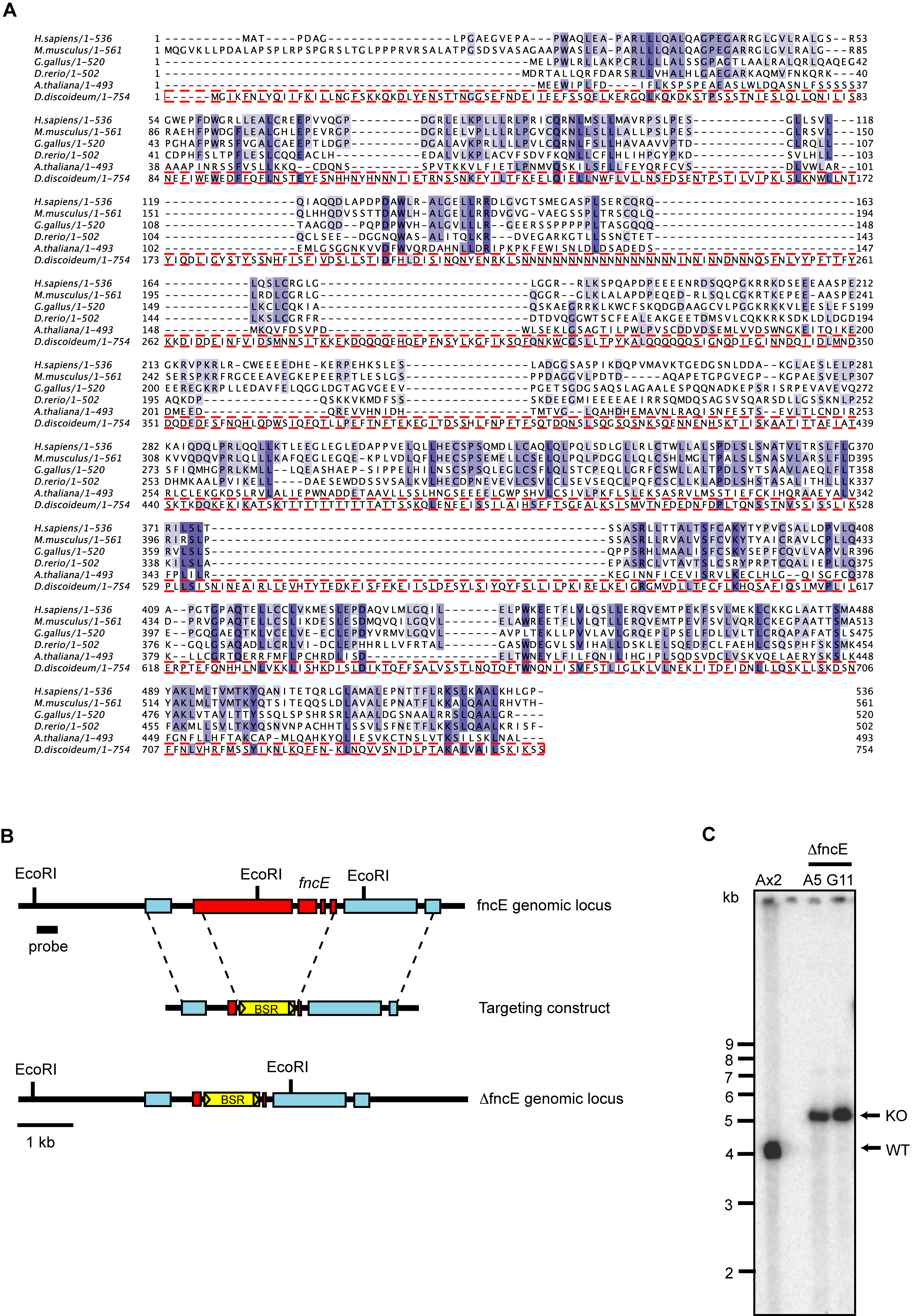

Supplement: Figure S4 — Generation and verification of the ΔfncE strain. (A) ClustalW alignment of the FncE sequences of Homo sapiens, Mus musculus, Gallus gallus, Danio rerio, Arabidopsis thaliana, and Dictyostelium discoideum. The Dictyostelium FncE sequence is highlighted by dashed red lines. (B) Generation and verification of the ΔfncE strain. Schematic representation of the targeting construct used for knocking out fncE (DDB_G0279669) and location of the probes and restriction sites used for Southern blot analysis. (C) Verification of the ΔfncE strain by Southern blot. This analysis resulted in a 4.0 kb band for WT cells and a 5.1 kb band for ΔfncE strains. (3.21 MB TIF) [file pgen.1000645.s004.tif]

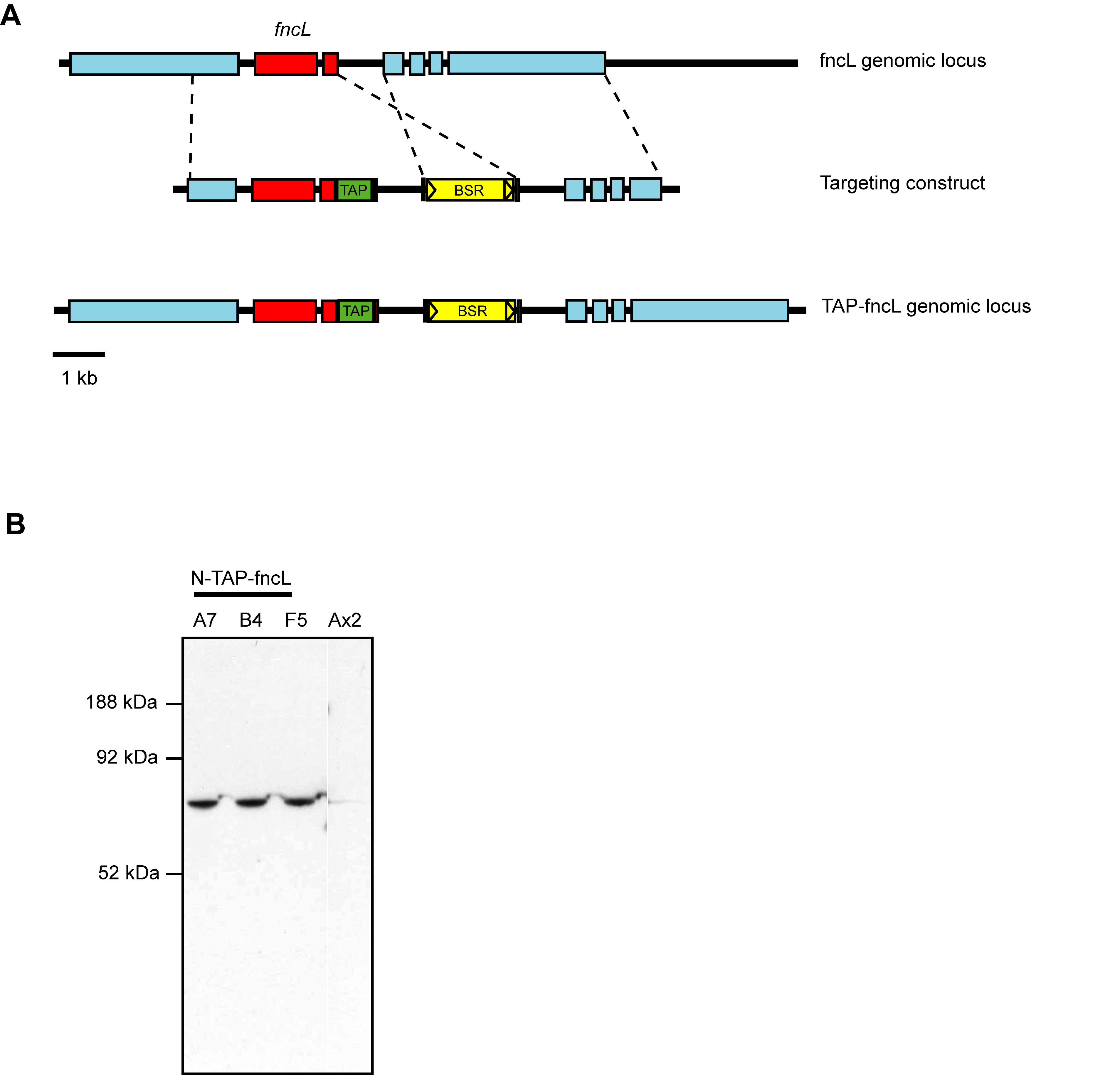

Supplement: Figure S5 — Generation and verification of the TAP-FncL strain. (A) Schematic representation of the targeting construct used for N-terminal in situ tagging of FncL with TAP. (B) Western blot showing TAP-FncL expression and specific detection of FANCL by the anti TAP antibody. Ax2 lysate was included as a negative control. The lysate of 7.5×10^5^ cells was loaded per lane. (1.35 MB TIF) [file pgen.1000645.s005.tif]

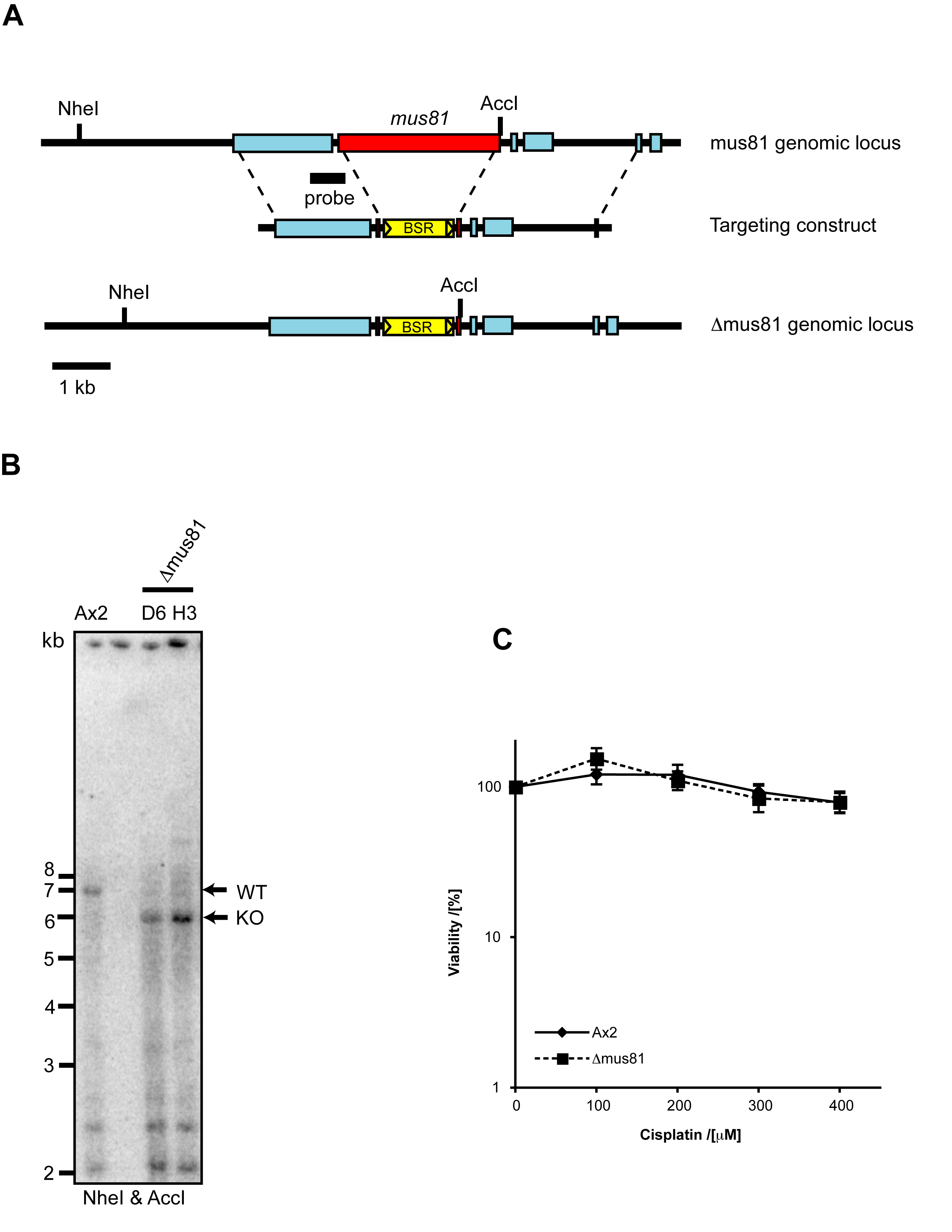

Supplement: Figure S6 — Generation and verification of the Δmus81 strain. (A) Schematic representation of the targeting construct used for knocking out mus81 (DDB_G0276519). (B) Verification of Δmus81 clones by Southern blot analysis. This analysis resulted in a 7.1 kb band for WT cells and a 6.0 kb band for Δmus81 strains. (C) The Δmus81 strain is not sensitive to cisplatin as assayed by colony survival. Results shown are from a single experiment. Error bars indicate variation between triplicate plating. (1.42 MB TIF) [file pgen.1000645.s006.tif]

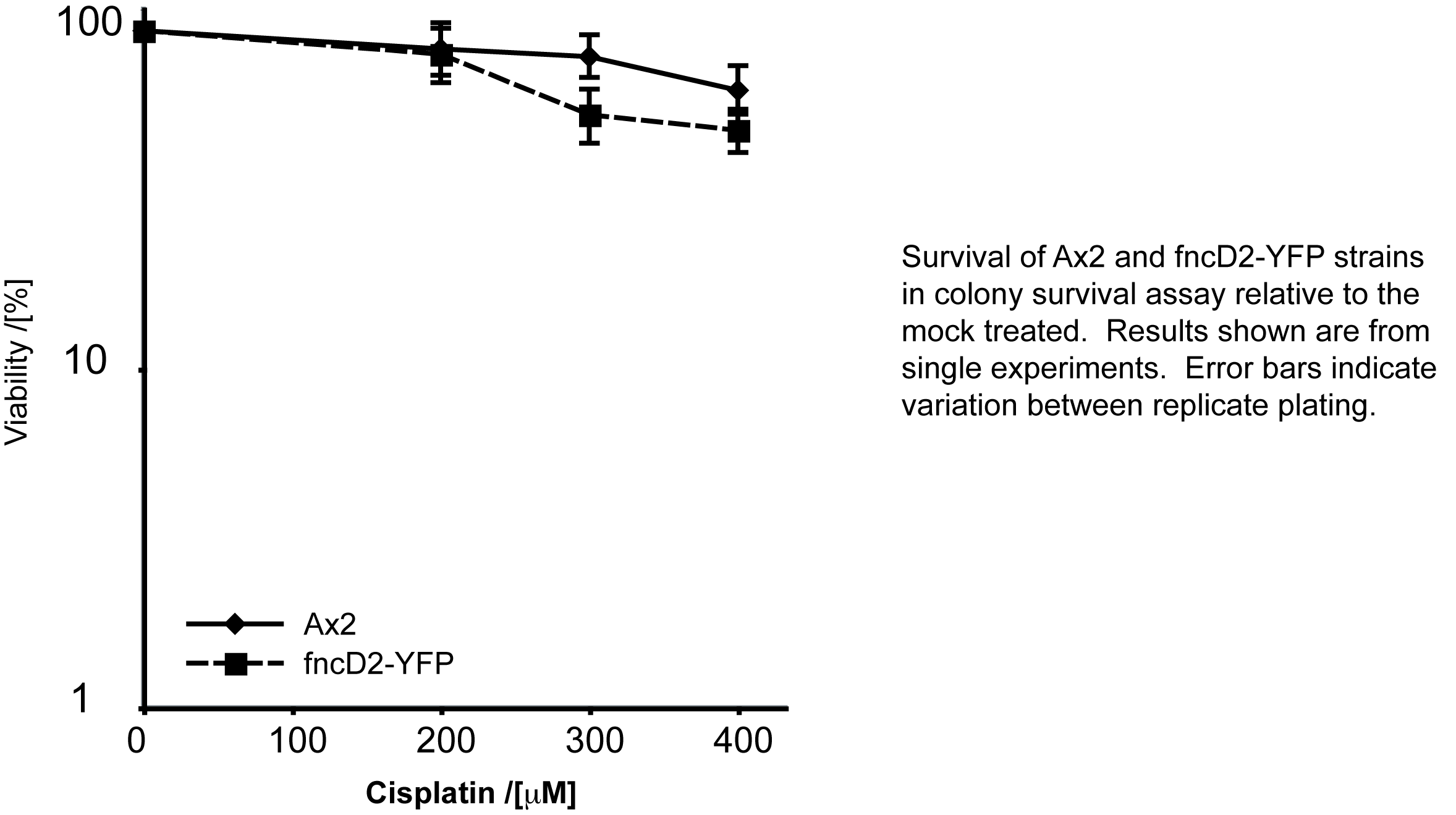

Supplement: Figure S7 — The FancD2-GFP strain is not sensitive to cisplatin. The FancD2 C-terminal GFP tagged strain does not show sensitivity to cisplatin. (0.29 MB TIF) [file pgen.1000645.s007.tif]
