# Supplementary material for: Xpf and Not the Fanconi Anaemia Proteins or Rev3 Accounts for the Extreme Resistance to Cisplatin in Dictyostelium discoideum
Source: PLoS Genet. 2009 Sep 18;5(9):e1000645. doi: 10.1371/journal.pgen.1000645 (PMC2730050; doi:10.1371/journal.pgen.1000645)
Supplement: Table S1 — Table of all the strains generated and used in this study. The systematic strain name (HMxxxx) is based on the nomenclature used in R. R. Kay's lab. Parental strain, genotype (Δ = deletion), overexpression plasmid present, and drug resistance of each strain are presented. (0.11 MB DOC) [file pgen.1000645.s008.rtf]

Supplementary Table 1: Genotype of strains generated in this study

Strain	Parent	Genotype	Overexpression	Selection	
HM1253	Ax2	fncD2		Blasticidin	
HM1302	Ax2	fncL		Blasticidin	
HM1303	Ax2	fncJ		Blasticidin	
HM1305	Ax2	fncD2-C-YFP		Blasticidin	
HM1306	HM1302 cl6-1	fncL cre-loxed		-	
HM1346	HM1307	ube2T fncD2-TAP		Blasticidin	
HM1348	HM1253 cl1	fncD2 cre-loxed		-	
HM1351	Ax2	rev3		Blasticidin	
HM1352	HM1348	fncD2 rev3		Blasticidin	
HM1356	Ax2	fncI		Blasticidin	
HM2559	HM1305	fncD2-C-YFP	pDXA-HA-ubiquitin	Blasticidin & G418	
HM1368	HM1306	fncL fncD2-YFP		Blasticidin	
HM1340	Ax2	N-TAP-fncL		Blasticidin	
HM2657	HM1368	fncL fncD2-YFP	pDXA-HA-ubiquitin	Blasticidin & G418	
HM2694	Ax2		pDXA-HA-ubiquitin	G418	
HM1403	Ax2	xpf		Blasticidin	
HM1405	HM1348	fncD2 xpf		Blasticidin	
HM1407	Ax2	fncM		Blasticidin	
HM1409	HM1305	fncD2-C-YFP cre-loxed		-	
HM1416	Ax2	fncE		Blasticidin	
HM1419	HM1409	fncE fncD2-YFP		Blasticidin	
HM2772	HM1419	fncE fncD2-YFP	pDXA-HA-ubiquitin	Blasticidin & G418	
HM1446	HM1351	rev3 E8 cre-loxed		-	
HM1447	HM1350	rev3 A10 cre-loxed		-	
HM1451	HM1447	rev3xpf		Blasticidin	
HM1458	Ax2	xpc		Blasticidin	
HM1463	Ax2	mus81		Blasticidin	
